# Recent Progress on Liver Kinase B1 (LKB1): Expression, Regulation, Downstream Signaling and Cancer Suppressive Function

**DOI:** 10.3390/ijms150916698

**Published:** 2014-09-19

**Authors:** Ren-You Gan, Hua-Bin Li

**Affiliations:** 1 School of Biological Sciences, The University of Hong Kong, Pokfulam Road, Hong Kong, China; E-Mails: ganry@connect.hku.hk; 2 Guangdong Provincial Key Laboratory of Food, Nutrition and Health, School of Public Health, Sun Yat-sen University, Guangzhou 510080, China

**Keywords:** liver kinase B1, LKB1, cancer, AMPK, MARK

## Abstract

Liver kinase B1 (LKB1), known as a serine/threonine kinase, has been identified as a critical cancer suppressor in many cancer cells. It is a master upstream kinase of 13 AMP-activated protein kinase (AMPK)-related protein kinases, and possesses versatile biological functions. *LKB1* gene is mutated in many cancers, and its protein can form different protein complexes with different cellular localizations in various cell types. The expression of LKB1 can be regulated through epigenetic modification, transcriptional regulation and post-translational modification. LKB1 dowcnstream pathways mainly include AMPK, microtubule affinity regulating kinase (MARK), salt-inducible kinase (SIK), sucrose non-fermenting protein-related kinase (SNRK) and brain selective kinase (BRSK) signalings, *etc.* This review, therefore, mainly discusses recent studies about the expression, regulation, downstream signaling and cancer suppressive function of LKB1, which can be helpful for better understanding of this molecular and its significance in cancers.

## 1. Introduction

The gene of liver kinase B1 (*LKB1*), encoding an approximately 50 kDa serine/threonine kinase, was first found mutated in Peutz-Jeghers Syndrome (PJS), which is a rare hereditary disease and is characterized by mucocutaneous pigmentation, increased risk of gastrointestinal hamartomatous polyposis as well as benign and malignant tumors [1,2,3,4]. In addition, germ-line mutations of LKB1 gene are also found in some extraintestinal cancers, such as lung cancer [5,6], breast cancer [7,8], cervical adenocarcinoma [5], pancreatic and biliary carcinoma [5,9,10], testicular cancer , malignant melanoma [11,12], head and neck squamous cell carcinoma [13] and hepatocellular carcinoma [14]. Besides, *LKB1* knockout or conditional knockout mice have been reported to develop abnormalities and tumors in many organs as shown in Table 1. These observations suggest that LKB1 has important functions, especially as a tumor suppressor.

**Table 1:** Abnormalities of *LKB1* knockout mice.

Model of Knockout Mice	Main Abnormalities	References
*LKB1*^−/−^ mice	Death at midgestation, with neural tube defects, mesenchymal cell death, and vascular abnormalities	[15]
*LKB1*^−/−^ mice	Death in utero between 8.5 and 9.5 days postcoitum, with developmental retardation of embryo	[16]
*LKB1*^+/−^ mice	Development of multiple gastric adenomatous polyps	[16]
*LKB1*^+/−^ mice	Development of hamartomatous polyps in gastrointestinal tract	[17]
*LKB1*^+/−^ mice	Development of hepatocellular carcinomas	[18]
*LKB1*^+/−^ mice	Development of severe gastrointestinal polyposis	[19]
Muscle-specific *LKB1*^−/−^ mice	Enhancement of insulin sensitivity in muscles	[20]
Endothelium-specific *LKB1*^−/−^ mice	Embryonic death at E12.5, with a loss of vascular smooth muscle cells and vascular disruption	[21]
Embryo fibroblasts *LKB1*^−/−^ mice	Defect of myofibroblast differentiation	[22]
*LKB1*(s) ^−/−^ mice	Male infertility, with abnormality at spermiation	[23]
*LKB1*^+/−^ mice	Development of osteogenic tumours	[24]
Prostate-specific *LKB1*^−/−^ mice	Development of prostate neoplasia	[25]
LKB1(s) ^−/−^ mice	Male infertility, with abnormal spermiation	[26]
Mullerian duct mesenchyme-derived cell-specific LKB1^−/−^ mice	Development of oviductal adenomas and endometrial cancer	[27]
Somatic testicular cells-specific *LKB1*^−/−^ mice	Male sub-infertility, with focal vacuolization in some of the seminiferous tubules, progressive germ cell loss and Sertoli cell only tubules	[28]
Adipose tissue-specific *LKB1*^−/−^ mice	Reduced amount of white adipose tissue, postnatal growth retardation, and early death before weaning	[29]
Muscle-specific *LKB1*^−/−^ mice	Reduced fatty acid oxidation during treadmill exercise	[30]
*LKB1*^−/−^ mice	Reduce of the latency of ErbB2-mediated tumorigenesis	[31]

Considering the importance of LKB1 in different cancers, LKB1 has attracted increasing interest for scientists, and LKB1-related researches have been rapidly increased. This review, therefore, summarized recent research progress about LKB1, including its expression, intracellular distribution, binding partners, regulation, downstream signaling pathways, and roles in cancer suppression. The above information of LKB1 can be helpful for better understanding of this molecular and its significance in cancers.

## 2. Expression Pattern of Liver Kinase B1 (LKB1)

Human *LKB1* gene, also known as serine/threonine kinase 11 (*STK11*), is located on chromosome 19p13.3 and composed of 10 exons spanning 23 kb [32]. It is identified to be the strong homolog of mouse *LKB1* gene, locating on mouse chromosome 10 and consisting of 10 exons covering about 15 kb [33], Xe*nopus laevis* egg and embryonic kinase 1 (*XEEK1*), *Caenorhabditis elegans* (*C. elegans*) partitioning defective gene 4 (*Par-4*) and *Drosophila LKB1* [34,35,36]. The mRNA of *LKB1* has been reported to exist splice variants, including mRNA length of 1302 and 444 bp in-frame deletion of exons 5–7 and part of exon 8, as well as variant retaining intron 4 [37,38]. *LKB1* gene is ubiquitously expressed in a variety of adult and fetal tissues and tumors. During mouse embryonic development, *Lkb1* mRNA is predominantly detected in gastrointestinal tract, lung and testis [39]. Similarly, human LKB1 is reported to be prominently expressed in epithelia and the seminiferous tubules of the testis, and expressed higher in fetal than in adult tissues [40]. In tumors, some malignant tumors express a high level of LKB1, but some cancer cells lost LKB1 expression, and this contradiction may be pertinent to the different process of tumorigenesis [40]. However, XEEK1 is reported to be apparently restricted to early embryogenesis [40], and Par-4 protein can be found in the gonads, oocytes and early embryos of *C. elegans* [35].

LKB1 protein functions as a serine/threonine kinase, and mutations of LKB1 proteins, such as K78I, D17GN, W308C, and L67P, lead to the loss of its kinase activity [32,41]. Murine Lkb1 shares about 90% identity with human LKB1 and has a conserved prenylation motif (Cys^433^–Lys–Gln–Gln^436^) at the carboxyl-terminus directly downstream from a consensus cAMP-dependent protein kinase (PKA) phosphorylation site (Arg^428^–Arg–Leu–Ser^431^) [42].

## 3. Subcellular Distribution of LKB1

LKB1 has different localizations in mammalian cells. In samples from PJS patients, wild type LKB1 is distributed in both the nucleus and cytoplasm, while the LKB1 mutant, SL26 (for amino acid deletion), has the normal kinase function, but only accumulates in the nucleus. Furthermore, the nuclear accumulation of LKB1 may be attributed to its amino terminal domain [43]. Similarly, *C. elegans* homolog Par4 is reported to be both cytoplasmically and cortically distributed [35]. When over-expressed in mammalian cells alone, LKB1 is mostly localized in the nucleus, with a small portion in the cytoplasm [33,44]. However, during the apoptosis of cells, LKB1 is found to translocate into mitrochondria [45,46]. In addition, when transfected with fused enhanced green fluorescent protein (EGFP)–LKB1 plasmid, LKB1 can be detected on both the plasma membrane and internal membranes *in vivo*, and this effect is mediated by a functional prenylation motif of LKB1 at the carboxyl-terminus, since mutation of Cys^433^ to an alanine residue of prenylation motif can block membrane localization of LKB1 [42]. It is also reported that LKB1 can form a complex between polyploidy-associated protein kinase (PAPK) and mouse protein 25 (MO25), resulting in the translocation of LKB1 from the nucleus to the cytoplasm and to tight junctions on the cell membrane [47]. Besides, when LKB1 is co-expressed with LKB1-interacting protein 1 (LIP1), a leucine-rich repeat containing cytoplasmic protein, the proportion of cytoplasmic LKB1 dramatically increases, and this indicates LIP1 may regulate LKB1 function by inducing its cytoplasmic localization [48].

## 4. Binding Proteins of LKB1

Endogenous LKB1 can mainly form two complexes which can regulate its stability and kinase activity. Through a yeast two-hybrid analysis and affinity purification from mammalian cells, LKB1 is found to form a heterotrimeric complex with two other proteins, termed STE20-related adaptor (STRAD) and MO25 [49,50,51]. STRAD, as a pseudokinase, can induce the transportation of LKB1 from the nucleus to the cytoplasm, and activate LKB1 kinase activity through promoting the active conformation of LKB1, while MO25, a 40 kDa scaffolding protein, directly binding to the conserved Trp–Glu–Phe sequence at the STRADα carboxyl-terminus, can markedly enhance the binding of STRAD to LKB1 and further stimulate STRAD-induced kinase activity of LKB1 [49,52,53].

On the other hand, LKB1 can also interact with a chaperone complex made up of heat-shock protein 90 (HSP90) and the CDC37, which can also stabilize LKB1 in the cytoplasm [54,55]. Furthermore, LKB1–HSP90–CDC37 complex is recently found to function as a repressor of LKB1 kinase activity and disruption of this complex facilitates HSP/HSC70 and E3 ubiquitin ligase carboxyl terminus of HSC70–interacting protein (CHIP)-mediated degradation of LKB1 [56]. Thus, LKB1–STRAD–MO25 and LKB1–HSP90–CDC37 complexes can both stabilize LKB1 with antagonizing effects on LKB1 activity. In addition, LKB1 is also reported to interact with other proteins, including LIP1, gene of phosphate and tension homology deleted on chromosome ten (PTEN), p53, brahma-related gene 1 (*BRG1*), activator of G-protein signaling 3 (AGS3), glycogen synthase kinase 3β (GSK3β), protein kinase ζ (PKCζ), ataxia telangiectasia mutated (ATM), as well as a complex with LIM domain only 4 (LMO4), GATA-binding factor 6 (GATA-6), and LIM domain-binding protein 1 (LDB1) [45,48,57,58,59,60,61,62].

## 5. Regulation of LKB1 Expression

### 5.1. Epigenetic Modification

Although the mutations of *LKB1* gene are found in several sporadic cancers, however, its somatic mutations are very rare. This indicates that the inactivation of *LKB1* gene may be also mediated by other mechanisms. Cancer cells may have abnormal patterns of DNA methylation including hypermethylation in gene promoter CpG islands and global demethylation of the genome. Studies found that hypermethylation of *LKB1* promoter region can be detected in different cancer cell lines and primary tumor samples. In several cancer cell lines, *LKB1* gene is found hyper-methylated at the CpG island in its promoter region, and correspondingly, *LKB1* transcript cannot be detected, while treatment with the demethylating agent 5-aza-2'-deoxycytidine can restore *LKB1* gene expression in these cells [46,63]. In addition, one primary colorectal carcinoma and three primary testicular tumors as well as parts of polyps from JPS patients display *LKB1* promoter hypermethylation but not in corresponding normal tissues [63]. It is consistent that in sporadic colorectal cancer, hypermethylation is found in the *LKB1* promoter as well [64]. These studies suggest that *LKB1* promoter methylation may be contributed to the silence of *LKB1* gene and LKB1-mediated functions.

### 5.2. Transcriptional Regulation

*LKB1* gene can be regulated by sex hormones, such as androgen and estrogen. Estrogen is reported to regulate *LKB1* gene expression through transcriptional regulation, and ERα binding site is found in the promoter region of *LKB1*. In MCF-7 breast cancer cells, 17β-estradiol can downregulate both mRNA and protein level of LKB1 through inhibiting *LKB1* promoter activity by reducing the binding of ERα to the promoter of *LKB1* [65]. Consistently, knockdown of ERα in MCF-7 cells can significantly increase *LKB1* promoter activity as well as LKB1 mRNA and protein levels [66]. However, estrogen is also reported to upregulate the expression of LKB1. In mouse and human adipocyte, 17β-estradiol can increase the mRNA level of LKB1 in a dose-dependent manner, and this effect is mediated by ERα, since PPT, an ERα-specific agonist, but not DPN, an ERβ-specific agonist, can increase the expression of LKB1 mRNA level [67]. Furthermore, ERα is found to bind to the region between −2287 and −2020 bp of the *LKB1* promoter and 17β-estradiol can significantly enhance the binding of ERα to the *LKB1* promoter [67]. In MCF-7 breast cancer cells, treatment of 17β-estradiol can increase the expression of LKB1, accompanied with reduced ERα expression [66]. Therefore, estrogen-mediated regulation of *LKB1* gene expression may be cell-context dependent.

In addition, androgen can also regulate the expression of LKB1 through the transcriptional level. In murine 3T3-L1 and human SGBS adipocytes, testosterone and dihydrotestosterone (DHT) can significantly decrease the mRNA level of LKB1, and this effect is mediated by androgen receptor (AR), since pre-treatment of flutamide, an AR antagonist, can block the effect of testosterone and DHT on LKB1 mRNA reduction [67]. Interestingly, although androgen can inhibit the mRNA level of LKB1 in adipocytes, there is no androgen receptor element (ARE) found in 2.5 kb promoter region of *LKB1* gene [67]. This indicates that AR may indirectly regulate the *LKB1* gene expression.

Besides, fibronectin can also regulate LKB1 expression. In non-small cell lung carcinoma (NSCLC) cells, fibronectin is found to inhibit both the mRNA and protein of LKB1 and LKB1–AMP-activated protein kinase (AMPK) signaling [68].

### 5.3. Posttranslational Modification

Researches find that LKB1 can be phosphorylated, prenylated and ubiquitinated in different conditions. Several kinases, such as PKA, PKC and ATM, have been reported to phosphorylate LKB1 protein. In *Drosophila* and murine, LKB1 has been reported to be phosphorylated by PKA [36,42]. In the perfused rat liver, glucagon can activate PKA, which then phosphorylates and activates LKB1–AMPK–mTOR signaling [69]. In immature rat granulosa cells, follicle stimulating hormone (FSH) can induce the Ser^428^ phosphorylation of LKB1, probably through AC–cAMP–PKA signaling [70]. PKCζ can activate LKB1 through phosphorylation of Ser^428^ of LKB1, resulting in binding of LKB1 with AMPK and subsequent AMPK Thr^172^ phosphorylation and activation [71]. LKB1 can also be phosphorylated at Ser^31^, Ser^325^ and Thr^366^ when expressed in HEK-293 cells, but its phosphorylation affects neither its nuclear localization nor its catalytic activity *in vitro* [72]. Other studies find that Thr^366^ of LKB1 can be phosphorylated by ATM [73], and activators of extracellular signal-regulated kinase 1/2 (ERK1/2) can induce the phosphorylation of endogenously expressed LKB1 at Ser^431^ [74]. In *Drosophila*, LKB1 can also be phosphorylated by Par1 *in vitro* [36].

The carboxyl-terminus of murine Lkb1 can be prenylated *in vivo*, and PKA-mediated LKB1 prenylation, but not phosphorylation, leads to membrane distribution of LKB1 [42]. Another study finds that in 293 cells, over-expressed LKB1 can be prenylated by addition of a farnesyl group to Cys^433^ [74].

Except phosphorylation and prenylation, LKB1 can be posttranslational modified by ubiquitination and deacetylation. The stability of LKB1 in cytoplasm is dependent on two protein complexes, LKB1–STRAD–MO25 and LKB1–HSP90–CDC37, and the latter complex can be disrupted by geldanamycin (GA), an HSP90-specific inhibitor, which can induce HSP70-CHIP-mediated degradation of LKB1 through the ubiquitination-proteasome pathway [56]. LKB1 can be deacetylated by SIRT1, which is a conserved NAD^+^-dependent deacetylase. SIRT1 functions as a negative regulator for LKB1/AMPK signaling in primary endothelial cells by promoting deacetylation, ubiquitination and proteasome-mediated degradation of LKB1 [75]. However, SIRT1 can also activate LKB1 by deacetylating and subsequently increasing the phosphorylation and activity of LKB1. In 293T cells, SIRT1 over-expression diminishes lysine acetylation of LKB1 and concurrently increases its activity and finally activated AMPK downstream signaling [76]. In HepG2 cells and mouse liver, over-expression of SIRT1 can stimulate basal AMPK signaling via phosphorylation and activation of LKB1 [77]. These results suggest that LKB1 is a target of SIRT1-mediated deacetylation, which can further influence its kinase activity and downstream signaling.

## 6. LKB1 Downstream Pathways

### 6.1. LKB1—A Master Kinase of AMPK-Related Protein Kinases

LKB1 is a master kinase of 13 AMPK-related protein kinases, including AMPK, NUAK family SNF1-like kinase 1 (NUAK1), sucrose non-fermenting protein-related kinase (SNRK), brain selective kinase1/2 (BRSK1 and BRSK2) or synapses of amphids-deficient kinase (SADK), Salt-inducible kinase1/2/3 (SIK1, SIK2 and SIK3), microtubule affinity regulating kinase1/2/3/4 (MARK1, MARK2, MARK3 and MARK4) or partitioning defective gene 1 (Par1) and maternal embryonic leucine zipper kinase (MELK) [78]. Except MELK, LKB1 can phosphorylate the T-loop of all other members and significantly enhance their kinase activities [78]. Furthermore, in LKB1-deficient cells, activities of endogenous NUAK2, SIKs and MARKs were significantly reduced [78]. Most AMPK-related kinases, such as SIK, MARK and BRSK isoforms, possess an ubiquitin-associated (UBA) domain at their carboxyl-terminus, close to the kinase catalytic domain. The UBA domain is found not to interact with polyubiquitin or other ubiquitin-like molecules, but may plays an essential conformational role and is required for LKB1-mediated phosphorylation and activation of their kinase activities, since mutation or removal of their UBA domains can markedly impair LKB1-induced phosphorylation and their catalytic activity [79].

### 6.2. LKB1–AMPK Signaling Pathway

AMPK is a critical serine/threonine kinase that functions as an intracellular energy sensor to maintain the energy balance within the cell. AMPK is a heterotrimeric protein complex containing a catalytic subunit α1/2 and two regulatory subunits, β1/2 and γ1/2. It is found that α subunit can directly interact with both β and γ subunits, however, whether β and γ subunits interact with each other remains controversial [80].

AMPK plays a central role in the regulation of whole-body energy metabolism, and AMPK activation is beneficial for protecting the body from metabolic diseases, such as type 2 diabetes and obesity. Mutations of AMPK can lead to cardiac hypertrophy and arrhythmia. Recent findings have identified that LKB1 is an important upstream kinase of AMPK cascade in mammalian cells [81,82]. LKB1 can phosphorylate Thr^172^ on the activation loop of AMPK catalytic subunit and subsequently activates AMPK *in vitro* [82,83]. In addition, inhibition of LKB1 activity in cells simultaneously abolishes the activation of AMPK by different stimuli [83]. Furthermore, LKB1-deficient murine embryonic fibroblasts show nearly complete loss of Thr^172^ phosphorylation and downstream AMPK signaling in response to different AMPK activators [82].

### 6.3. LKB–MARK/Par1 Signaling Pathway

Mammalian MARKs are the homolog of *C. elegans* Par-1. They were first purified from brain and can phosphorylate microtubule associated proteins (MAPs), such as MAP2, MAP4 and tau, resulting in their dissociation from microtubules [84]. The subcellular distribution of MARK isoforms has some difference. Wild-type MARK1 and MARK3 have a similar subcellular distribution, and both are present in the cytoplasm and plasma membrane, whereas the 17A-MARK3 mutant, in which all 17 phosphorylation sites of MARK3 have been converted to alanine residues, is strictly localized at the plasma membrane, and MARK3 membrane localization needs a highly conserved carboxyl-terminal domain [85]. MARK2 is reported to be localized to the plasma membrane to a larger degree than MARK3, and over-expression of wild-type PKCζ can induce a marked increase of wild-type MARJ2 in the cytoplasmic localization, but cannot cause significant change of the subcellular localization of the T508A-MARK2 mutant, MARK1, MARK3 and MARK4 [85]. In addition, MARK4 is observed to be localized to filamentous structures in confocal microscopy images [85].

LKB1 is an important upstream kinase of MARKs/Par-1, and LKB1-mediated activation of MARKs has been reported to regulate microtubule dynamics. In *Drosophila*, LKB1 is required for the phosphorylation and activation of PAR-1, which in turn promotes tau phosphorylation [86]. Diverse stress stimuli, such as high osmolarity and over-expression of the human β-amyloid precursor protein, can promote PAR-1 activation and tau phosphorylation in an LKB1-dependent manner [86]. In addition, LKB1 can phosphorylate and activate MARK2, which in turn phosphorylates microtubule-associated protein Tau at the KXGS motif and suppresses tubulin polymerization [87]. Consistently, over-expression of LKB1 in cells suppresses microtubule regrowth, while knockdown of LKB1 accelerates it [87]. Furthermore, the phosphorylation of Tau by the LKB1-MARK signaling triggers proteasome-mediated degradation of Tau [87]. These studies indicate that LKB1-MARK signaling is a critical regulator of the cellular microtubule cytoskeleton.

### 6.4. LKB1–SIK Signaling Pathway

SIK1 and SIK2/QIK are members of AMPK-related kinases. SIK1 is a regulator in the feedback cascades of cAMP-mediated gene expression. It can be activated by LKB1 through phosphorylation of Thr^182^ and Ser^186^, which is essential for its kinase activity [88]. LKB1–SIK–TORC signal has been reported to inhibit the activity of cAMP response element-binding protein (CREB), which is a transcription factor that can activate transcription when its Ser^133^ is phosphorylated. Transducer of regulated CREB activity (TORC) is a transcriptional coactivator of CREB and can upregulate CREB activity in the nucleus. SIK is reported to inhibit CREB activity through TORC. SIK can interact with and phosphorylate TORC, leading to the nuclear export of TORC [89,90]. However, in LKB1-defective HeLa cells, SIK is unable to phosphorylate TORC, which causes constitutive activation of CRE activity, and this effect is not mediated by CREB phosphorylation at Ser^133^ [90]. Similarly, in LKB1-deficient hepatocyte, TORC2 is dephosphorylated and enters the nucleus, inducing the expression of peroxisome proliferator-activated receptor-γ coactivator 1α (PGC-1α)—a master coordinator of mitochondrial biogenesis—and in turn drives gluconeogenesis [91]. In addition, knockdown of TORC2 can reduce PGC-1α expression and normalize blood glucose levels in *LKB1*-knockout mice, indicating that TORC2 is a critical target of LKB1 in the regulation of gluconeogenesis [91]. These researches indicate that LKB1–SIK signal plays an important role in silencing CREB activity via phosphorylation and nuclear export of TORC, and such silencing may regulate CREB target genes.

### 6.5. LKB1–SNRK/NUAK2 Signaling

SNRK/NUAK2 is a member of the AMPK-related protein kinases and can be activated by LKB1 through phosphorylation of the Thr^173^ residue at its T-loop region, and the LKBI regulatory subunits STRAD and MO25 are necessary for LKBI-mediated activation of SNRK/NUAK2 [92]. SNRK/NUAK2 has been detected in the thymus, spleen, kidney and stomach, with higher levels found in the liver, skin, testis, uterus and ovary and the greatest levels in the adrenal and brain tissue, but the with highest kinase activity in the testis [93]. It is predominantly localized in the nucleus, probably through a conserved nuclear localization signal (NLS) at the amino-terminus. Deletion and point mutation of this part result in the cytoplasmic translocation of mutant SNRK/NUAK2 proteins. In addition, the nuclear localization of SNRK/NUAK2 can modulate gene expression profile [94]. Besides, myosin phosphatase target subunit 1 has been found to be the only substrate of NUAK2 to date [95].

### 6.6. LKB1–BRSK/SADK Signaling

BRSK1/SADK-B and BRSK2/SADK-A are members of AMPK related kinases that are highly expressed in mammalian forebrain [96]. In addition, they are also slightly expressed in the testis and pancreas. They can be activated by LKB1 through phosphorylation of Thr^174^ in the T-loop activation segment of their kinase domain [96]. On the other hand, protein phosphatase 2C is a likely candidate for catalyzing the dephosphorylation and inactivation of BRSK1/2 [96]. In the mammalian cerebral cortex, LKB1-BRSK/SADK signaling is reported to be involved in the polarization of neurons. LKB1 Ser^431^ phosphorylation by PKA or p90RSK results in BRSK/SADK phosphorylation and activation, which then phosphorylates MAPs that implement polarization [97].

## 7. LKB1 Functions as a Cancer Suppressor

**Figure 1:**
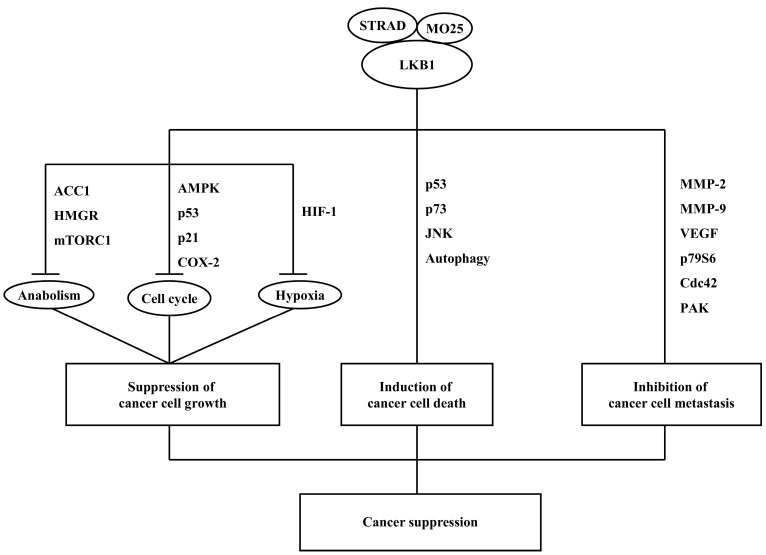
A diagram describing the potential mechanisms of liver kinase B1 (LKB1)-mediated cancer suppression.

LKB1 is mutated in many cancer cells, and further investigations demonstrated that LKB1 is a cancer suppressor. It has been reported that LKB1 and its downstream signaling cannot only suppress cancer cell growth or induce cancer cell death, but also can inhibit cancer cell metastasis. The potential mechanisms of LKB1-mediated cancer suppression are summarized in Figure 1.

### 7.1. Suppression of Cancer Cell Growth

LKB1 has been reported to inhibit the growth of cancer cells, but not normal cells, and this may be partly due to various stresses in cancer cells, such as metabolic stress and hypoxia. Under metabolic stress, such as energy deprivation, LKB1-AMPK signaling can be activated in cancer cells [98]. AMPK activation can subsequently inhibit anabolism and induce cell cycle arrest, and finally suppress cancer cell growth.

LKB1 inhibits anabolism of cancer cells through AMPK activation. LKB1–AMPK signaling can inhibit fatty acid synthesis through suppression of acetyl-CoA carboxylase 1 (ACC1), inhibit sterol synthesis through suppression of 3-hydroxy-3-methylglutaryl-CoA reductase (HMGR) and inhibit protein synthesis through suppression of the mammalian target of rapamycin (mTOR) complex 1 (mTORC1) [98].

On the other hand, LKB1 can induce cell cycle arrest of cancer cells through AMPK signaling. In cancer cell lines, AMPK activators, such as metformin, can significantly inhibit cell growth by induction of cell cycle arrest through AMPK activation, and this effect may be associated with suppression of anabolism through inhibition of mTOR by phosphorylating tuberous sclerosis 2 (TSC2) [99,100,101]. Besides, AMPK can also initiate cell cycle arrest through persistent phosphorylation and activation of p53, which leads to accelerated p53-dependent cellular senescence [102]. In addition, LKB1 may inhibit cancer cell growth through AMPK-selective cyclooxygenase-2 (COX-2) signaling. Inhibition of COX-2 can induce cancer cell cycle arrest and growth inhibition. Activation of AMPK has been reported to decrease COX-2 expression in colon cancer cell lines, and AMPK is found to be an upstream signal to inhibit COX-2 expression and COX-2-mediated cell proliferation [103]. Importantly, over-expression of wild-type LKB1 is reported to down-regulate the mRNA and protein level of COX-2 in lung cancer cells [104]. On the other hand, ectopic expression of cyclooxygenase-2 can inhibit LKB1 activity in MCF-7 cells [105]. Therefore, these results indicate that LKB1–AMPK–COX-2 signaling is involved in inhibition of cancer cell growth and also has a negative feedback effect.

Besides, LKB1 can induce cell cycle arrest of cancer cells dependent on p21 and p53. LKB1-mediated G1 cell cycle arrest is caused by up-regulation of the expression of CDK inhibitor p21^WAF1/CIP1^, and this effect can be antagonized by co-expression of the G1 cyclins—cyclin D1 and cyclin E [44,106]—and these effects are p53-independent [62]. On the other hand, LKB1 was also reported to physically interact with p53 in the nucleus to stabilize p53, and directly or indirectly phosphorylate p53 Ser^15^ and Ser^392^, which are required for LKB1-dependent G1 cell cycle arrest [106]. These results indicate that LKB1 has a direct role in activation of *p21^WAF1/CIP1^* gene.

In addition, LKB1 may inhibit cancer cells growth through regulation of hypoxia inducible factor 1 (HIF-1) under hypoxic condition. Hypoxia is an important feature in most cancers [107]. Hypoxia can induce the expression of (HIF-1), which is a transcription factor up-regulated under hypoxic condition, and HIF-1 can subsequently activate genes that permit cancer cells to survive and grow in the hypoxic tumor environment [108]. It has been well established that LKB1-AMPK signaling can negatively regulate mTORC1 [109], and HIF-1 protein has been reported to be up-regulated by mTORC1 [110]. In addition, in LKB1-deficient tumors *in vivo*, mTORC1 and HIF-1 have been reported to be drastically up-regulated [111]. These results indicate that LKB1-AMPK-mTORC1 signaling may inhibit the expression of HIF-1 and its target genes responsible for cell survival, and finally suppress cancer cell growth under hypoxic condition.

Finally, LKB1 can inhibit cancer cell growth with other mechanisms. Endogenous STRAD knockdown can abrogate G1 cell cycle arrest, which indicates that STRAD plays a crucial role in the tumor suppressor effect of LKB1 [49]. In addition, LKB1 can interact with and activate BRG1, which can subsequently induce cell growth arrest [58].

### 7.2. Induction of Cancer Cell Death

It is found that in polyps derived from PJS patients, there is a lack of LKB1 staining and reduced numbers of apoptotic cells, and LKB1 is a mediator of p53-dependent cell death [45]. However, in the pancreatic cancer cell line AsPC-1, LKB1-induced cell apoptosis is reported to be p53-independent, but through p73-dependent cell death [46]. In addition, LKB1 can induce cell apoptosis through activation of JNK pathway [112]. Besides, in osteosarcoma cells, LKB1 is critical for TRAIL and death associated protein 3 (DAP3)-induced cell apoptosis, while K78M LKB1, a kinase dead mutant, can inhibit DAP3-induced cell apoptosis in these cells [113].

However, LKB1 can also induce autophagy, which has opposite effects on the fate of cancer cells. On one hand, autophagy plays a predominant role in sustaining the survival of cancer cells with defects in apoptosis. On the other hand, autophagy can also induce cell death under some conditions. Under metabolic stresses, such as hypoxia and energy deprivation, LKB1-AMPK signaling can be activated, and subsequently phosphorylates and activates tuberous sclerosis complex 2 (TSC2), which next inhibits mTOR and mTORC1, leading to the activation of autophagy [114]. Whether LKB1-controlled autophagy in cancer cells can induce cell survival or cell death remains elusive, and it may be dependent on cell types and tumor micro-environmental condition, such as metabolic stress.

### 7.3. Inhibition of Cancer Cell Metastasis

Research has also found that LKB1 can inhibit cancer cell invasion and metastasis. In MDA–MB-435 breast cancer cells without LKB1 expression, over-expression of wild-type LKB1 can significantly inhibit the invasion and metastasis of cancer cells *in vitro* and *in vivo*, and this is accompanied with downregulation of matrix metalloproteinase 2 and 9 (MMP-2 and MMP-9) as well as vascular endothelial growth factor (VEGF) [115]. Similarly, LKB1 is essential for adiponectin-mediated inhibition of migration and invasion of breast cancer cells, and this effect is dependent on AMPK activation and p70S6 kinase inhibition [116].

### 7.4. Therapeutic Approaches Targeting LKB1

Since LKB1 has been well demonstrated to be a cancer suppressor in many cancers, pharmacological approaches targeting LKB1 downstream signaling may be used for cancer treatment. Indeed, several agents targeting LKB1 downstream signaling, including AMPK activators, mTOR and COX-2 inhibitors, have been reported to be effective for some cancers, the representatives of which and their effects on cancer cells are listed in Table 2.

**Table 2:** Representative agents targeting LKB1 signaling associated with cancer treatment.

Drugs	Target of LKB1 Signaling	Effects on Cancer Cells	References
Celecoxib	Inhibition of COX-2	Inhibition of polyps *in vivo*	[117]
Metformin	Activation of AMPK	Induction of cancer cell death	[118]
Rapamycin	Inhibition of mTOR	Suppression of tumor *in vivo*	[119]
Resveratrol	Activation of AMPK	Inhibition of caner cell growth	[120]

### 7.5. LKB1—Still a Cancer Promoter?

Although LKB1 has been well demonstrated to be a cancer suppressor, recent studies also report some paradoxical results, and imply that under specific conditions, LKB1 can be a cancer promoter. For example, under energy-deprived conditions, LKB1-deficient fibroblasts have been reported to exhibit a greater degree of cell death compared to wild-type cells [82]. In addition, in LKB1-deficient lung adenocarcinoma (A549) cells, over-expression of LKB1 plasmid is found to fight against glucose starvation-induced cancer cell death, and this effect is mediated through inhibition of fatty acid synthesis by AMPK activation [121]. Besides, knockdown of LKB1 downstream molecular AMPK and NUAK1 in osteosarcoma (U2OS) cells with over-expression of c-Myc can lead to cell apoptosis [122]. More recently, it is reported that phenformin, an AMPK activator, can be more effective in the treatment of non-small cell lung cancer (NSCLC) if the tumors lack a functional LKB1-AMPK pathway [123]. These researches indicate that in some cell types, LKB1 signaling can normally protect cancer cells from metabolic stresses or oncogene over-expression. Therefore, considering the promoting cancer effect of LKB1, care should be taken when targeting LKB1 signaling for cancer treatment.

## 8. Conclusions and Perspectives

As a master kinase of AMPK-related kinases, LKB1 plays important pathophysiological functions in different diseases, especially in cancers. This review summarized recent advances about LKB1 researches, and mainly discussed its expression pattern, intracellular distribution, binding proteins, regulation manners, main downstream signaling pathways, and roles in cancer suppression. Although LKB1-AMPK signaling has been extensively investigated in cancer cells, whether other LKB1 downstream pathways, such as LKB1-MARK/SIK/SNRK pathways, are also involved in LKB1-mediated cancer suppressive effect remains unclear. In addition, latest researches indicate that LKB1 can also regulate cell polarity and cell integrity, which play a critical role in cancer cell metastasis, and whether LKB1-mediated regulation of cell polarity can affect the metastasis of cancers needs further investigation.
